# Interprofessional approach to personalized medication management and therapy optimization in IBD care

**DOI:** 10.3389/fmed.2025.1446695

**Published:** 2025-01-29

**Authors:** Daniel Fleischmann, Benedicta Binder, Muriel Huss, Tanja Elger, Claudia Wolf, Johanna Loibl, Hauke Christian Tews, Arne Kandulski, Stephan Schmid, Martina Müller, Alexander Kratzer

**Affiliations:** ^1^Hospital Pharmacy, University Hospital Regensburg, Regensburg, Germany; ^2^Department of Internal Medicine I, Gastroenterology, Hepatology, Endocrinology, Rheumatology, and Infectious Diseases, University Hospital Regensburg, Regensburg, Germany

**Keywords:** inflammatory bowel disease, patient safety, clinical pharmaceutical care, interprofessional collaboration, medication management

## Abstract

A considerable number of patients with chronic inflammatory bowel diseases (IBD) are required to manage extensive polypharmaceutical regimes, which significantly elevates the risk of drug–drug interactions. Also, the disease’s impact often leads to the consumption of additional self-medication by the patients such as naturopathic remedies to alleviate disease-induced suffering and nutritional supplements to compensate for malabsorption syndromes inherent to the condition. There is a well-established consensus that polymedication coupled with unregulated supplementary intake can jeopardize the safety of drug therapy. Despite this, pharmaceutical co-supervision—proven to mitigate adverse drug events and enhance patient adherence to treatment—is generally lacking in routine clinical settings. Furthermore, the assessment of individual therapy adherence, a crucial predictive factor for therapeutic outcomes, is frequently suboptimal. In response to these issues, this study implemented an interdisciplinary approach wherein a team comprising medical and pharmaceutical professionals conducted a comprehensive survey coupled with a medication review for patients attending an IBD outpatient clinic. Employing an IBD-specific questionnaire alongside the patients’ documented medication regimens enabled the identification and subsequent discussion of current therapeutic concerns and potential medication-related risks during follow-up consultations. This intervention aimed to bolster individual patient satisfaction and enhance medication safety, ultimately fostering sustained success in IBD management.

## 1 Introduction

Inflammatory bowel diseases (IBD), comprising Crohn’s Disease (CD) and Ulcerative Colitis (UC), represent a complex array of pathologies characterized by both acute and chronic inflammation within the gastrointestinal tract. Current estimates suggest a prevalence rate of 0.3%, with a global uptrend in incidence attributable to various factors, underscoring the growing relevance of IBD in contemporary and future internal medicine (1–4). Recent pharmacological advancements have expanded the therapeutic arsenal, notably with the inclusion of target-specific antibodies, which are playing an increasingly pivotal role in IBD treatment (5, 6). Nonetheless, the management of IBD, whether newly diagnosed or pre-existing, poses substantial challenges due to numerous obstacles faced by patients and healthcare providers throughout the therapeutic process.

A significant aspect of IBD is its considerable psychological impact, as it is a chronic condition requiring long-term management. Psychosocial stressors can critically influence disease progression (7–9). Although psychological co-supervision is recognized as a beneficial component of comprehensive IBD care, many patients seek additional self-directed interventions with non-prescription drugs. In that regard, Bauer et al. found in a recent nationwide German survey, that 50% of IBD patients were using complementary or alternative medicines (CAM) in self-medication alongside their actual IBD medication (10). Additionally, Lakatos et al. found that the use of CAM was more common in patients undergoing supportive psychiatric/psychological therapy. CAM also encompass naturopathic remedies, such as plant-derived products, which carry a notable risk of hepatotoxicity, as well as specialized nutritional supplements, including multivitamin preparations that frequently contain potentially hazardous concentrations of certain components (11). While it is understandable for IBD patients to look for such supplementary approaches which could be potentially advantageous, they also introduce risks, such as undermining treatment efficacy or precipitating additional health issues due to adverse drug interactions. This is especially problematic given the high incidence of polypharmacy among IBD sufferers and its associated risks. In this context, a recent retrospective study by Mesonero et al. found, that 18.4% of all surveyed patients were simultaneously using 5 or more drugs, a threshold that is commonly used to define polypharmacy. The authors also concluded that polypharmacy was mainly found in older adults and those with comorbidities and that it was the only factor associated with IBD treatment nonadherence in the study (12).

In this context, enhanced pharmaceutical co-supervision could play a critical role in improving medication safety and efficacy while also bolstering compliance with established treatment regimens (13–16). Adequate adherence to therapy is a key determinant of successful long-term management of IBD (17, 18). Despite widespread recognition of its importance, patient adherence is rarely evaluated through systematic assessments (19).

To address these gaps, our study deployed an interdisciplinary team of physicians, nurses, and specialized clinical pharmacists at an IBD outpatient clinic. This team leveraged an existing interprofessional network within the Department of Internal Medicine to conduct a multi-faceted evaluation and optimization of patient-specific medication strategies (20, 21). The primary objective was to integrate assessments of individual treatment adherence (i.e., the reliable intake of prescribed IBD medication as well as regular appearance to scheduled appointments in our clinic) with comprehensive pharmaceutical reviews to identify and mitigate drug-related issues and optimize therapeutic outcomes. Moreover, the study aimed to offer a more personalized, multidisciplinary approach to treatment, thereby promoting more consistent adherence to therapy. Additionally, it sought to uncover individual and systemic barriers to effective IBD management within the outpatient setting, facilitating improvements in patient care and treatment success.

## 2 Methods

### 2.1 Study design

This prospective, transversal, unicentric, open-label study was conducted at the Department of Internal Medicine I (specializing in Gastroenterology, Hepatology, Endocrinology, Rheumatology, and Infectious Diseases) of the University Hospital Regensburg, Germany, from April 1 to December 31, 2023.

### 2.2 Study population

Participants included both male and female patients aged 18 years or older who either had a confirmed diagnosis of IBD per the European Crohn’s and Colitis Organization (ECCO) criteria or presented symptoms indicative of inflammatory gastrointestinal disorders pending an IBD diagnosis (22, 23). Exclusion criteria encompassed individuals with an insufficient understanding of the questionnaires as well as incomplete medical records and/or questionnaires.

### 2.3 Procedure

Three weeks prior to their scheduled visit at the IBD outpatient clinic, eligible patients received an invitation by mail or e-mail to partake in the study through completion of a preliminary questionnaire detailing their current therapy regimen and medication plan (Supplementary Figures S1, S2). This questionnaire was mainly based on previously existing questionnaires that had been used in our clinic to assess patients´ medication and therapeutic adherence and was subsequently modified for this IBD study using various pre-existing, standardized tests assessing therapy adherence and IBD patient experience (24–26). The questionnaire featured sections on diagnosis history, adherence levels, personal understanding of their therapy, and open-ended items querying additional counseling needs. Responses were gauged on a 5-point Likert scale (27). Subsequently, a clinical pharmacist evaluated the provided medication plan and initial questionnaire to identify areas for therapeutic enhancement or potential adherence obstacles. The review process emphasized drug dosage, interaction risks, and possible adverse effects of the medications. Non-indicated medications were flagged for discontinuation, and adjustments to therapy based on international IBD guidelines were recommended where applicable (22, 23).

If participants reported using nutritional or plant-based supplements, these substances were critically assessed for efficacy and safety through comprehensive literature reviews. Outcomes of these pharmaceutical reviews were systematically documented and categorized (28). Findings were then collaboratively discussed with the attending physicians before patient consultations, allowing for informed adjustments to therapeutic approaches based on the insights gathered.

### 2.4 Post-consultation follow-up

Following their clinic visits, patients completed a second questionnaire focusing on their personal experiences and the potential impact of the initiative on pre-existing concerns over their therapy and on the individual knowledge about their IBD medication (Supplementary Figure S3).

### 2.5 Statistical analysis

**Figure 1:**
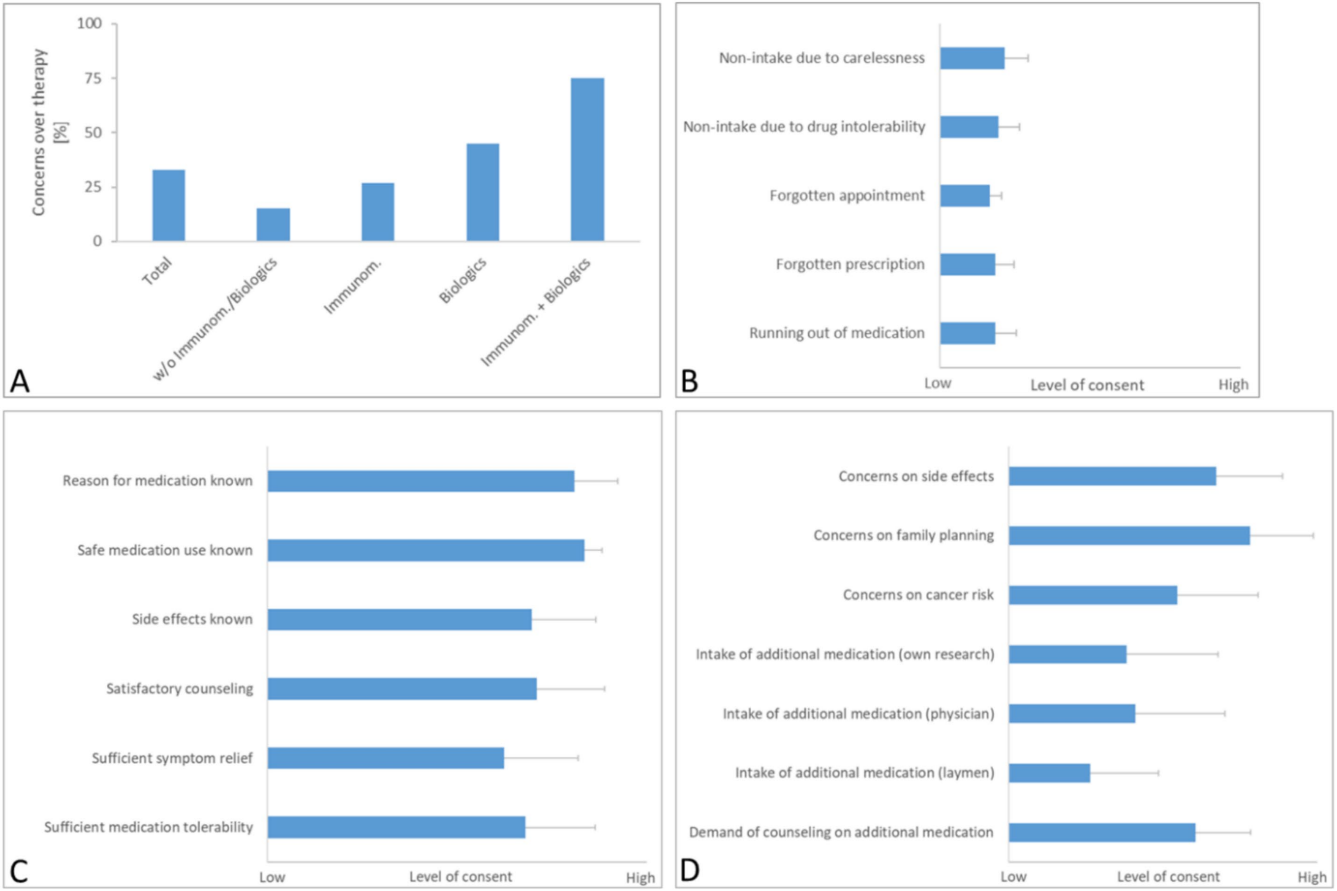
Results of questionnaire 1. Interviewees were asked to answer questions on general concerns over their therapy **(A)**, the pre-existing therapy adherence **(B)** and their current medication knowledge and satisfaction **(C)**. Also, respondents could indicate, whether they had specific concerns over their medication and if they were taking additional drugs or supplements for their IBD therapy **(D)**. Results are depicted as mean with SD (*N* = 42). For results depicted in panels **(B–D)**, a Likert scale from 1 (no consent) to 5 (high consent) was used. For the question on concerns over implications for their family planning **(D)**, merely answers from patients younger than 45 years were analyzed (*N* = 27).

**Figure 2 fig2:**
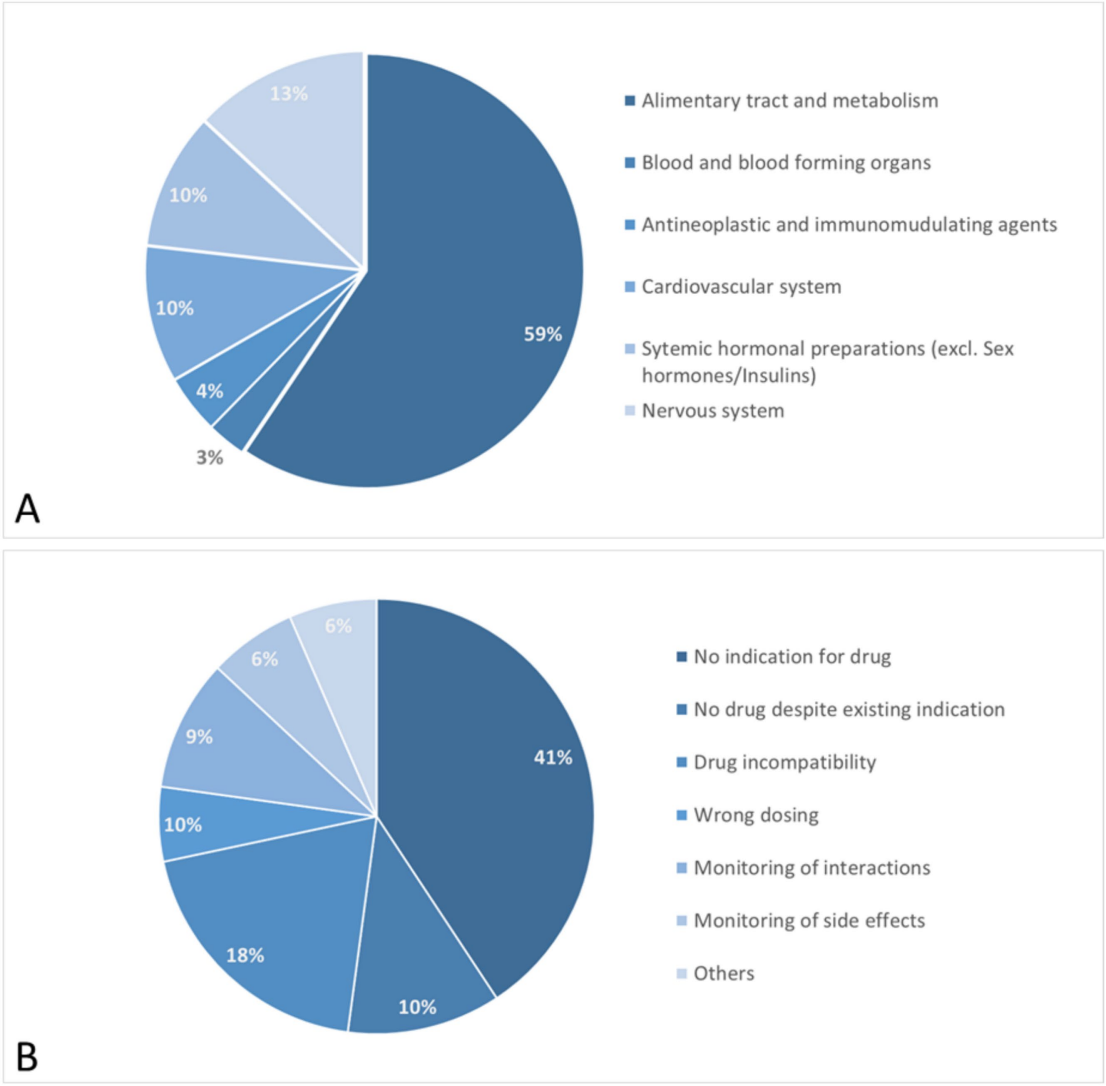
Results of pharmaceutical medication check. ATC classification of affected drugs **(A)** and documented pharmaceutical interventions **(B)**.

Demographic and disease-specific data were summarized in terms of absolute (n) and relative (%) frequencies (Table 1). The pharmaceutical intervention outcomes were likewise quantified (Table 2). Questionnaire responses and medication review data were analyzed, presenting averages and standard deviations (Figures 1–3).

**Table 1:** Demographic and disease characteristics of patients (*N* = 42).

**Median age (range) - years**	48 (18–74)
Male	52
Female	46
Sex	
Male	13 (31%)
Female	29 (69%)
Diagnosis	
Crohn’s Disease (CD)	16 (38%)
Ulcerative Colitis (UC)	15 (36%)
Inclassificable Colitis	11 (26%)
Initial diagnosis	
<1 yr	6 (14%)
1–5 yrs	12 (29%)
5–10 yrs	6 (14%)
>10 yrs	18 (43%)
Treatment in outpatient clinic	
<1 yr	12 (29%)
1–5 yrs	16 (38%)
5–10 yrs	2 (4%)
>10 yrs	12 (29%)
Disease activity	
In remission	18 (43%)
Mild to moderate symptoms	24 (57%)
Severe symptoms	0
Medication	
No IBD Medication/5-ASA/Corticosteroids	7 (15%)
Immunomodulators	15 (36%)
Azathioprin	7 (16%)
Cyclosporin A	4 (10%)
Tacrolimus	4 (10%)
Biologics	20 (48%)
Infliximab	7 (17%)
Adalimumab	6 (14%)
Vedolizumab	3 (7%)
Ustekinumab	4 (10%)

**Table 2:** Overview of pharmaceutical interventions after medication check (*N* = 42).

First medication check	36 (86%)
Degree of pharm. intervention	33 (79%)
Polymedication (>5 drugs)	30 (71%)
Pharm. interventions per patient	1.6 (±0.4)
Degree of implementation	92% (61 of 66 pharm. interventions)
Discontinuation of medication	9 (21%)
New medication started	6 (14%)

**Figure 3:**
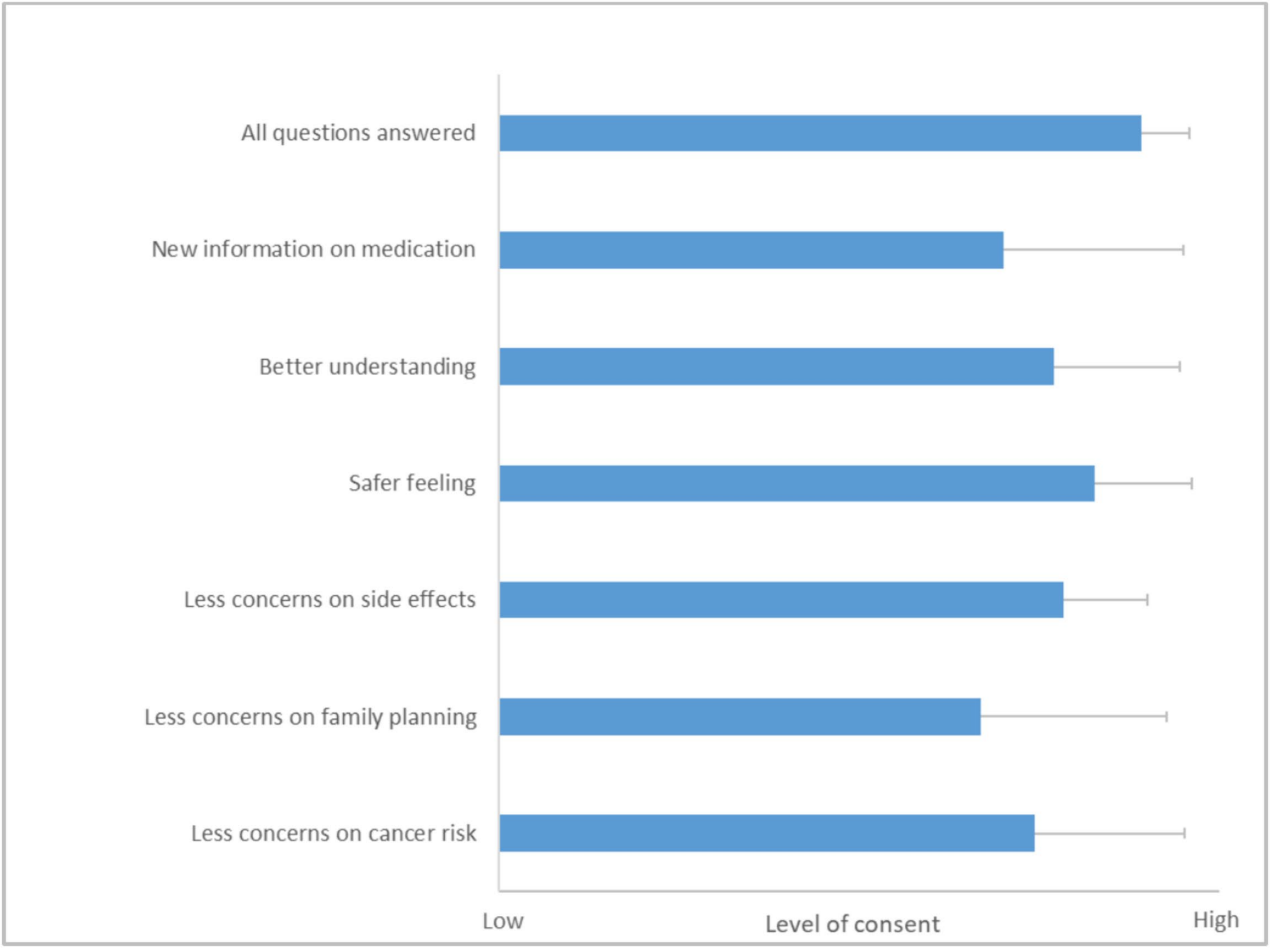
Results from questionnaire 2 on client experience and implications on future therapy adherence. A Likert scale from 1 (no consent) to 5 (high consent) was used. Results are depicted as mean with SD (*N* = 42). For the question on concerns over implications for their family planning, merely answers from patients younger than 45 years were analyzed (*N* = 27).

### 2.6 Ethical considerations

The study adhered to the ethical standards of the University Hospital Regensburg’s human research guidelines. Ethical approval was secured prior to the commencement of the study. Participants provided informed consent, ensuring the anonymity and confidentiality of their data. All participant information derived from questionnaires and medication reviews was anonymized to prevent any identification of individual patients.

## 3 Results

During the study, 97 patients were invited to partake in the study and data was fully collected from 42 patients at the IBD outpatient clinic. The median age of the cohort was 48 years, with a predominance of female participants, who constituted 69% of the sample (Table 1). The distribution of diagnoses within the group showed a near balance between CD and UC, with 38 and 36%, respectively. Additionally, 11 patients were categorized under a preliminary diagnosis of inclassificable colitis, indicating a potential form of inflammatory bowel syndrome pending a definitive diagnosis.

The historical data on diagnosis and therapy revealed that a significant portion of the patients had a longstanding relationship with the clinic. Specifically, 43% of the patients reported having received their initial diagnosis over 10 years ago, and 29% had started their IBD treatment at this outpatient clinic during the same time frame. This long-term engagement highlights the chronic nature of IBD and the extended duration of care that is often required. According to the medical documentation after the appointment in the outpatient clinic, 43% of surveyed patients were in clinical remission at the date of their appearance in the outpatient clinic while 57% of all participants reported mild to moderate symptoms. There was no patient with severe symptoms. Disease activity was thereby assessed according to the ECCO guidelines (22, 23).

Regarding the therapeutic regimens reported, a significant number of patients were on complex medication plans involving advanced pharmacological treatments. These included immunomodulators such as azathioprine, cyclosporine A, and tacrolimus, as well as biologic therapies. Biologics used by the patients included anti-TNF-*α* agents (infliximab, adalimumab), integrin blockers (vedolizumab), and interleukin blockers (ustekinumab). These treatments reflect the current standards in IBD management, targeting various pathways to reduce inflammation and manage symptoms in severe cases. Only 15% of all surveyed patients did not take any specific IBD medication or merely aminosalicylates (5-ASA) or corticosteroids at the time of the survey.

### 3.1 Therapy adherence and individual concerns

#### 3.1.1 Concerns about current therapy

The initial segment of the questionnaire revealed that 33% (14/42) of all respondents harbored general concerns about their ongoing IBD therapy (Figure 1A). Notably, the expression of concern was disproportionately higher among patients prescribed with immunomodulatory substances (27%, 4/15) and biologics (45%, 9/20). Specifically, only 15% of patients (1/7) not on such medications (i.e., patients currently taking no specific IBD medication or merely aminosalicylates or corticosteroids) expressed doubts about their therapy, whereas a significant 75% (3/4) of those on a combination of immunomodulators and biologics reported concerns, reflecting apprehensions possibly tied to the complex side effects and long-term implications of these potent drugs.

#### 3.1.2 Adherence based on reliable intake of prescribed medication and appearance at medical appointments

In the second part of the questionnaire (Figure 1B), the majority of patients reported to consistently take their medication as prescribed and to diligently arrange for prescription refills and medical appointments.

#### 3.1.3 Knowledge and information satisfaction

Responses regarding knowledge of medication and its impact on symptom control (Figure 1C) showed that most patients were well-informed about the indications and dosing of their medications. Additionally, a substantial number reported satisfaction with the information provided about potential side effects, although the level of satisfaction regarding symptom relief and medication tolerability was slightly lower but still substantial, scoring 3.4 and 3.7 out of 5 on the Likert scale, respectively.

#### 3.1.4 Additional concerns and consultation needs

In alignment with earlier findings, a significant number of participants expressed heightened concerns about side effects like increased cancer risk and the implications of treatment on family planning (the latter assessed only among patients under 45 years of age) (Figure 1D). Regarding the use of non-prescription medications, such as plant-derived drugs and dietary supplements, about 50% of the respondents indicated that they were using these products, based on their own research or the advice of a healthcare provider. Interestingly, reliance on recommendations from non-medical sources was less common.

Moreover, a notable portion of the cohort voiced a desire for further guidance on additional medications or supplements during upcoming visits, highlighting an ongoing need for comprehensive patient education and support in managing their IBD therapy effectively.

#### 3.1.5 Pharmaceutical medication check

As part of the study, patients were required to submit their current medication regimen, which could either be in the form of a nationally harmonized medication plan or a personal list that included all medications and supplements being used. Surprisingly, 86% of participants reported that they had never undergone a medication check of the kind performed in this study (Table 2).

The data also revealed that a substantial 71% of patients were taking more than five different drugs, surpassing the threshold commonly associated with polymedication, which is frequently correlated with an elevated risk of medication errors and adverse drug interactions (29–31).

Following the review of the submitted medication lists, a potential pharmaceutical intervention was identified for 79% of all medications analyzed. On average, 1.6 interventions were documented per patient. Notably, 61 of the 66 recommended pharmaceutical changes (92%) were implemented by the attending physicians, indicating a high level of collaboration between the pharmaceutical and medical staff.

Furthermore, for 35% of the patients, the pharmaceutical review led to either the initiation of a new medication or the discontinuation of an existing one.

During the study, a comprehensive medication review highlighted various drug classes impacted by pharmaceutical interventions. Notably, the majority of interventions involved medications for the alimentary tract and metabolism, which represented 59% of all drugs reviewed. This category includes treatments for acid-related issues, functional gastrointestinal disorders, bile and liver therapies, antidiarrheals, medications for constipation, supplements, vitamins, and antidiabetic drugs.

Furthermore, 13% of the interventions targeted drugs affecting the nervous system, such as analgesics, antiepileptics, antiparkinsonian drugs, and psycholeptics.

Cardiovascular medications also accounted for 10% of the interventions, encompassing antihypertensives, diuretics, vasodilators, vasoprotectives, beta and calcium channel blockers, drugs affecting the renin-angiotensin system, and lipid-modifying agents.

Additionally, systemic hormonal preparations, including systemic corticosteroids, thyroid hormones, and pancreatic hormones, were also adjusted in 10% of the cases.

The specific pharmaceutical interventions revealed several key insights into the management of medication plans among IBD patients. A significant 41% of the cases involved medications for which no clear indication was found either in the medication plan or the patient record. This lack of indication highlights a substantial area of concern where drugs may be prescribed without sufficient documentation or justification, emphasizing the need for rigorous review and justification of each medication’s use.

In 23% of these cases, these interventions focused on naturopathic remedies and nutritional supplements mainly comprising herbal products containing curcuma or artichoke extracts and various combinations of b vitamins. For these products used in self-medication, no clear medical indication could be established in most instances. Additionally, these products often carry a risk of severe adverse effects, such as acute liver injury. In 10% of the cases, the introduction of an additional drug was advised to manage a pre-existing condition, suggesting that some patients’ current treatment regimens were insufficient to fully address their medical needs.

Furthermore, 18% of the intervention instances pertained to drug incompatibilities where the concomitant use of multiple drugs led to significant physico-chemical interactions. These interactions could drastically reduce the bioavailability of the involved medications, potentially compromising treatment effectiveness.

Optimization of the dosing regimen, including adjustments to the total dose and dosing intervals, was required in another 10% of the cases.

Finally, monitoring for potential pharmacological drug interactions (involving CYP450 enzyme system and others) and possible side effects was recommended in 9 and 6% of cases, respectively.

In the follow-up questionnaire, patients shared their experiences of the appointment at the outpatient clinic, reflecting on how it influenced their therapy adherence and overall satisfaction. The results from this feedback were overwhelmingly positive (Figure 3). Most patients felt that the discussion during their appointment was comprehensive, with a majority rating the coverage of all relevant topics at an impressive 4.9 out of 5.0.

Further insights from the questionnaire showed that the patients gained valuable knowledge about their medication during these sessions. They reported a better understanding of their therapy, scoring an average of 4.2 out of 5.0, which highlights the informative nature of the consultation. Additionally, the clarity and depth of the information provided seemed to enhance their sense of security regarding their treatment, as evidenced by a safety feeling score of 4.6 out of 5.0.

A significant aspect of the consultation was its impact on the patients’ perceptions and possible concerns about their medication. The majority thereby noted a decrease in concerns about potential side effects, with a score of 4.3 out of 5.0. This improvement is crucial as it likely contributes to higher adherence and better overall management of their condition. The patients also indicated that discussions during the appointment alleviated worries about the effects of their therapy on family planning and the risk of developing cancer.

## 4 Discussion

The outcomes of the questionnaires and comprehensive medication checks conducted during the study offer significant insights into how patients perceive their current IBD therapy and how they adhere to prescribed treatments.

A significant proportion (33%) of study participants reported substantial concerns about their current IBD therapy. This observation is consistent with findings from a recent study, wherein participants expressed notable concerns, particularly regarding their treatment and its potential side effects. In that study, a visual analog scale (VAS) ranging from 0 to 100 was employed to assess these concerns. Among *n* = 113 patients aged 35–59, concerns about medication effects were rated at a mean value of 65 on the VAS (32). In our study, individual concerns were markedly more pronounced among patients prescribed immunomodulatory substances and biologics, with such individuals exhibiting two to three times more concern than those not receiving these treatments. The heightened apprehension reached a peak in patients receiving combination therapy of both drug classes, with 75% expressing concerns, although it’s important to note that the number of patients in this specific subgroup was relatively small (*N* = 4).

The study’s findings emphasize the intricate interplay between advanced therapeutic strategies for IBD—which frequently necessitate the use of potent pharmacological agents associated with considerable adverse effects—and the consequential psychological burden experienced by affected patients (33, 34). This underscores the critical need for healthcare providers to consider both the physical and emotional well-being of patients when planning and administering treatment. These findings have been incorporated into national and international clinical guidelines, which emphasize the importance of considering both clinical efficacy data and the potential psychological burden on patients when selecting and implementing potent therapies for IBD (35, 36). In our outpatient clinic, we therefore closely collaborate with the Department of Psychosomatics of our university hospital to ensure optimal patient care.

The study revealed a high degree of consistency by the patients in taking their medications and staying engaged with their healthcare providers, with data mirroring findings from a previous survey by Bager et al., which reported an overall therapy adherence rate of 93% among IBD outpatients (37). The respondents also showed a commendable level of knowledge concerning why and how to take their medications, and the potential side effects involved. However, despite their good understanding, many patients still harbored significant concerns about the long-term implications of their medications, particularly in relation to adverse drug reactions, the potential risk of cancer, and effects on family planning. These fears seem to stem from a comprehensive awareness of the chronic nature of their condition and the lifelong dependency on medication it entails.

A considerable portion of the patients also expressed a need for more in-depth counseling about potential additional medication options for treating IBD, as well as a notable number of patients taking additional medication or nutritional supplements based on their own research. This proactive approach to self-management highlights a gap in the patient-provider communication that could be bridged with more thorough and frequent pharmaceutical counseling sessions.

In terms of the medication checks performed during the study we could find three main areas of interest:

Firstly, a significant majority of the interventions involved medications for the digestive tract and metabolism, reflecting the intricate medication regimens that IBD patients often must navigate. This finding emphasizes the challenge of managing a disease that not only affects the gastrointestinal system directly but also requires careful balancing of nutritional needs and medication effects.
Secondly, more than half of the interventions resulted from either inappropriate medication use without a clear indication or a missing medication that was indicated. In 35% of these cases, this led to either the introduction of new medications or the discontinuation of existing ones. This high rate of medication modification underscores the complexity of IBD symptoms and the involvement of multiple healthcare providers, which can sometimes lead to fragmented care without adequate coordination.
Lastly, the significant desire for more detailed counseling on additional medication options reveals the deep psychological impact of chronic diseases like IBD. This need for more information also showcases the potential of pharmaceutical counseling to enhance patient treatment satisfaction and outcomes.

Overall, the integration of pharmaceutical counseling into routine IBD care was highly valued by patients, as evidenced by improved understanding and satisfaction with their treatment following these sessions. This proactive approach not only meets the immediate clinical needs but also significantly enhances the treatment experience, fostering better health outcomes and adherence to therapies. Such findings advocate for a more integrated, patient-centered approach in managing IBD, emphasizing the importance of addressing both the medical and emotional needs of patients.

## 5 Conclusion

In the management of IBD, the complexity inherent in polymedication and escalated therapy regimens can significantly hinder patient adherence. To address these challenges and enhance patient satisfaction and safety in outpatient settings, our interdisciplinary team undertook a comprehensive evaluation of current treatment practices. This involved the implementation of a dual-part approach: administering a detailed questionnaire to assess patient perceptions and concerns regarding their therapy, and conducting a thorough pharmaceutical medication check to evaluate and optimize their current treatment regimens.

The data collected from these initiatives revealed telling insights. A notable percentage of patients expressed substantial concerns about their ongoing treatments, highlighting a pervasive sense of unease and uncertainty about the long-term effects and efficacy of their medication regimes. Many patients indicated a desire for more in-depth discussions about potential additional treatment options, suggesting a need for broader information dissemination and more personalized treatment planning.

Furthermore, the medication checks performed revealed ample opportunities for optimization of current treatment regimens. In many instances, adjustments made to the medications not only aligned better with best practice standards but also addressed individual patient needs more effectively, reducing the risk of adverse drug reactions and enhancing the overall treatment efficacy. In this context, we strongly advocate for the implementation of a routine medication review during every visit for IBD patients. Such an initiative holds the potential to enhance patient safety substantially and optimize therapeutic outcomes, thereby addressing critical aspects of patient care in this population. Furthermore, this approach could be highly beneficial in more effectively integrating the patient perspective into clinical practice (38).

The outcomes of this interdisciplinary approach have been highly positive, resulting in significant improvements in patient satisfaction and medication safety. It is important to acknowledge, however, that the overall number of interviewees was not particularly high and only from one outpatient clinic, and it is likely that primarily patients with an already elevated level of adherence agreed to participate in the study. Also, long term effects on patients´ therapeutic adherence could not be adequately measured with our study design, as the time span of the survey and intervention would have been too short. Finally, the IBD-specific questionnaire that was used in this study, needs to be further validated with a greater number of participants to guarantee reproducibility and consistency among larger numbers of interviewees. Despite these limitations, the positive feedback from patients clearly highlighted the value of personalized and attentive care. This tailored approach not only enhanced patient satisfaction but also considerably increased trust in the treatment process, emphasizing its importance in clinical practice. As a result of these successes, our team plans to intensify these efforts moving forward, continuing to refine and expand our methods to ensure that every patient receives the most effective and safe treatment possible. This initiative not only supports better health outcomes but also encourages a more engaged and informed patient community, essential for long-term disease management in IBD.

## Data Availability

The original contributions presented in the study are included in the article/Supplementary material, further inquiries can be directed to the corresponding author.
