# Supplementary material for: Interprofessional approach to personalized medication management and therapy optimization in IBD care
Source: Front Med (Lausanne). 2025 Jan 29;12:1446695. doi: 10.3389/fmed.2025.1446695 (PMC11814462; doi:10.3389/fmed.2025.1446695)
Supplement: Supplementary file 1 [file Table_1.docx]

**- Supporting information -**

**Interprofessional Approach to Personalized Medication Management and Therapy Optimization in IBD Care**

D. Fleischmann^1^, B. Binder^2^, M. Huss^2^, T. Elger^2^, C. Wolf^2^, J. Loibl^2^, H.C. Tews^2^, A. Kandulski^2^, S. Schmid^2^, M. Müller-Schilling^2^, A. Kratzer^1^.

^1^University Hospital Regensburg, Hospital Pharmacy, Regensburg, Germany

^2^University Hospital Regensburg, Department of Internal Medicine I- Gastroenterology- Hepatology- Endocrinology- Rheumatology and Infectious diseases, Regensburg, Germany.


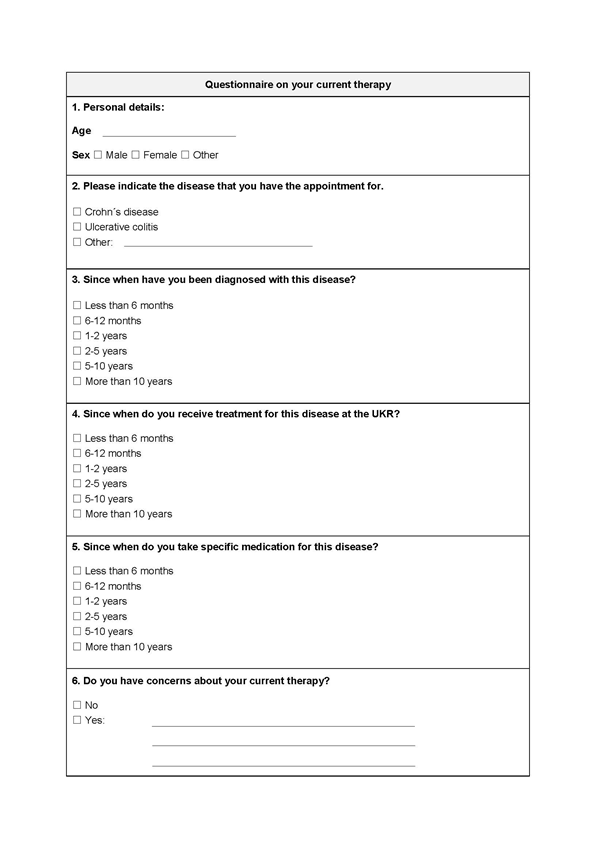


**Figure S1**. Questionnaire 1 – Personal details on diagnosis and previous therapy.


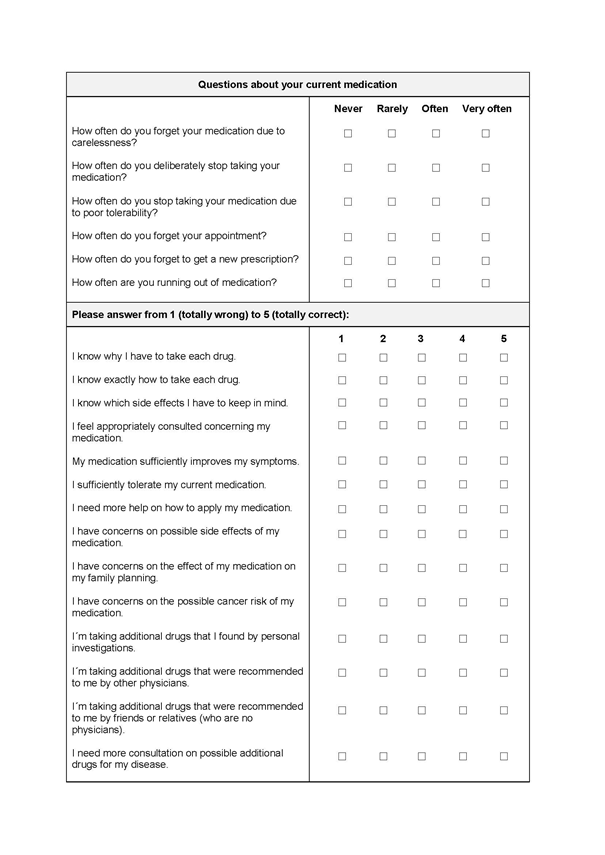


**Figure**

 **S2.** Questionnaire 1 – Questions on therapy adherence, individual knowledge and need for consultation.


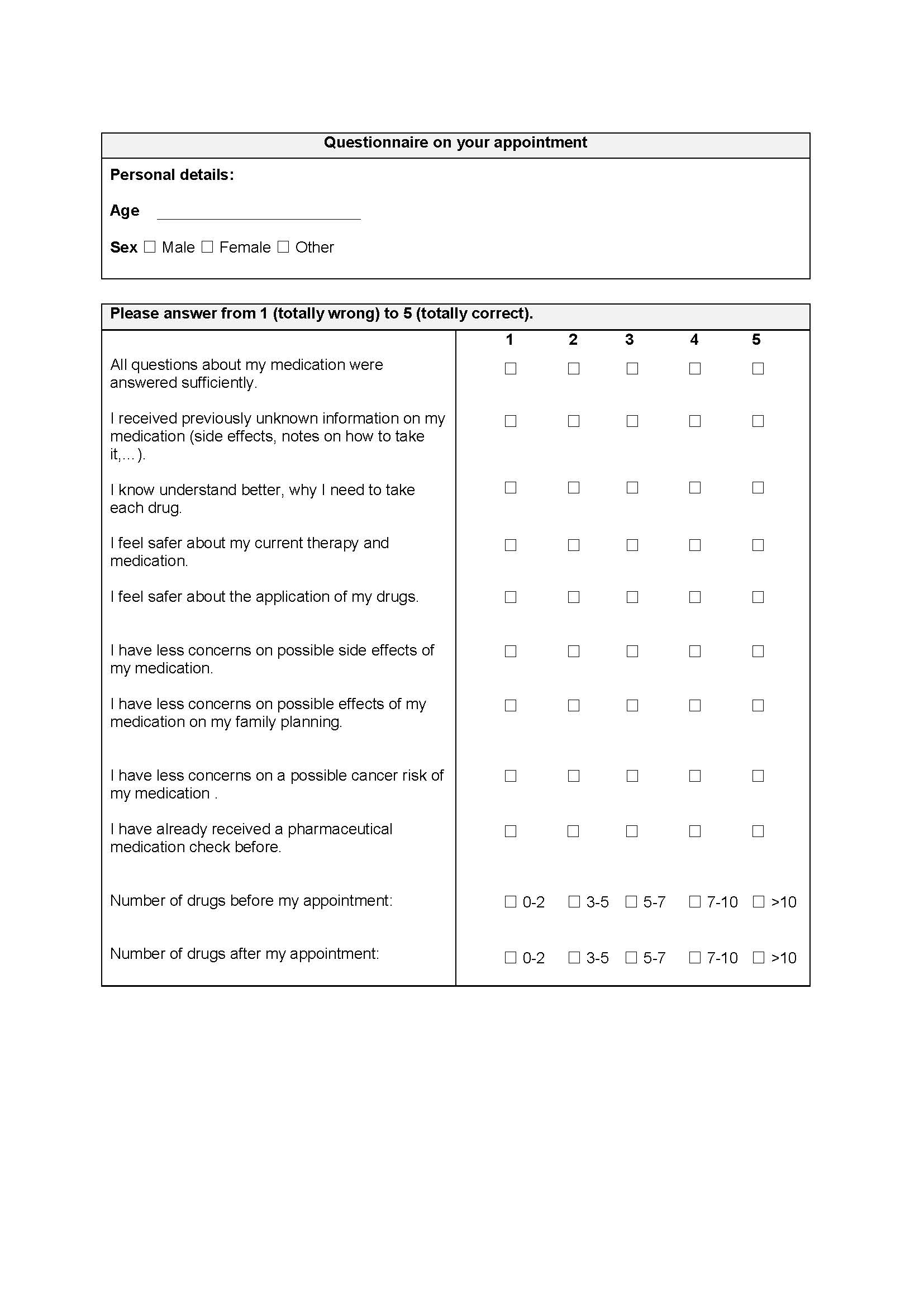


**Figure S3.** Questionnaire 2 – Questions on client experience and further therapy adherence.
